# Anti-Inflammatory Effects of *Ajuga decumbens* Extract under Blue Light Stimulation

**DOI:** 10.4014/jmb.2502.02002

**Published:** 2025-04-24

**Authors:** Tae-Jin Park, Byeong Min Choi, Hyehyun Hong, Jin-Soo Park, Seung-Young Kim

**Affiliations:** 1 Department of Pharmaceutical Engineering & Biotechnology, Sunmoon University, Asan 31460, Republic of Korea; 2 Natural Product Informatics Research Center, Korea Institute of Science and Technology, Gangneung 25451, Republic of Korea

**Keywords:** *Ajuga decumbens*, LED, anti-inflammatory, plant callus, MAPK

## Abstract

We investigated the potential of *Ajuga decumbens* Thunb. as an anti-inflammatory agent by utilizing plant resources to develop materials through the application of tissue culture and light-emitting diode (LED) cultivation technologies. To compare the changes between *A. decumbens* callus extract (ADCB) cultivated under blue monochromatic LED light and ADC (the negative control) cultivated under dark conditions, their morphological characteristics were tested and LC/MS analyses were conducted. ADCB exhibited a greenish hue compared with ADC and contained increased levels of specific compounds. The anti-inflammatory activities of the two samples were evaluated using LPS-stimulated macrophages. None of the samples exhibited cytotoxicity at any tested concentration. However, ADCB demonstrated a greater ability to reduce nitric oxide and key pro-inflammatory cytokines including interleukin-1β, interleukin-6, and tumor necrosis factor-α compared to the control. Furthermore, ADCB effectively suppressed the expression of inducible nitric oxide synthase and cyclooxygenase-2. It inhibited the phosphorylation of mitogen-activated protein kinase family proteins, including extracellular signal-regulated kinase, c-Jun N-terminal kinase, and p38, in a concentration-dependent manner. Tissue culture and LED cultivation technologies are significant methods for addressing plant supply challenges and enhancing the content of bioactive compounds, thereby increasing the applicability of plant materials. Moreover, ADCB produced using these technologies exhibited anti-inflammatory activity without causing irritation to human skin at active concentrations, suggesting its potential as a novel anti-inflammatory material.

## Introduction

The inflammatory response is an innate immune reaction involving immune cells that remove and regenerate tissues damaged by external stimuli, such as pathogens or toxins, and defend against invading factors [1, 2]. Lipopolysaccharide (LPS), which is present in the outer membrane of gram-negative bacteria, binds to CD14, an LPS-binding protein in host cells, including macrophages, and subsequently activates Toll-like receptor 4, which triggers the mitogen-activated protein kinase (MAPK) signaling pathway. This activation is known to induce the expression of inflammatory cytokines, such as tumor necrosis factor-a (TNF-a), interleukin-1β (IL-1β), and interleukin-6 (IL-6), as well as inflammatory mediators like inducible nitric oxide synthase (iNOS) and cyclooxygenase-2 (COX-2) [3].

The MAPK signaling pathway consists of extracellular signal-regulated kinase 1/2 (ERK), p38 MAPK, c-JUN N-terminal kinase (JNK). It plays a critical role in regulating physiological responses by transmitting extracellular stimuli, such as cytokines, into intracellular signals and is essential for cancer response and resistance [4, 5]. ERK1/2 transmits signals related to cell proliferation and differentiation, and its excessive activation induces abnormal cell proliferation, leading to cancer [6]. JNK and p38 regulate cell survival, apoptosis, inflammatory and stress responses, and expression of inflammatory cytokines [7-9].

Nitric oxide (NO), a representative inflammation-related factor, is synthesized by nitric oxide synthase. Of the three isoforms of nitric oxide synthase, neuronal and endothelial NOS are constitutively expressed in neuronal and endothelial cells, respectively, whereas iNOS is expressed in stimulated macrophages and is involved in inflammation [10, 11]. COX-2 is an inducible enzyme that converts arachidonic acid into prostaglandin (PG) during inflammatory responses, contributing to pain, fever, and inflammation. Unlike COX-1, which is normally expressed in various tissues, COX-2 promotes PG synthesis upon stimulation, thereby triggering inflammatory responses [12]. Prostaglandin E_2_ (PGE_2_), a representative PG that exacerbates inflammation, is known to cause edema and pain [13]. Although inflammation induced by these processes is essential in the body, excessive production of reaction-inducing factors due to external stimuli can result in chronic diseases, such as cancer, arthritis, atherosclerosis, and neurological disorders, as well as nerve damage [14, 15]. Therefore, to prevent and effectively treat inflammatory diseases, extensive research is being conducted on anti-inflammatory agents focusing on natural products with few side effects and high stability [16-18].

Plants of the genus *Ajuga* have traditionally been used as remedies for fever and infections and exhibit various physiological activities, including antimalarial, anti-inflammatory, and antioxidant effects, making them valuable medicinal plants [19]. Certain species contain ecdysteroids that serve as natural biodegradable insect repellents [20]. Among them, *A. decumbens* Thunb. (AD), which is distributed in temperate regions of Asia and Europe, has been reported to exhibit biological activities, such as anti-inflammatory, anticancer, anti-hyperlipidemic, and antibacterial effects [21-23]. Furthermore, it contains diterpenes and iridoid glycosides, which have been shown to be effective in treating chronic pelvic inflammation and uterine fibroids [24, 25]. However, despite their diverse biological activities and potential as functional materials, the use of these natural plant resources is limited by seasonal restrictions, environmental variability, and challenges in stable supply, necessitating alternative production methods [26].

Plant tissue culture techniques are gaining attention as potential solutions for these issues. Callus culture refers to the formation of undifferentiated cell masses under specific environmental conditions or after cell damage. These cells, which lack specialized functions but continuously divide and proliferate, have been used in various studies [27]. Callus cultures are advantageous because they can be cultivated indoors, enable the production of genetically identical plants and secondary metabolites on a large scale, and play a crucial role in preserving endangered plants, genetic modifications, and plant cloning, making them highly valuable for biotechnological research and industrial applications [28].

Recently, research has been actively conducted on the effects of controlling the light environment using light-emitting diodes (LEDs) on plant growth and metabolism. LEDs, which were first developed in the United States in the 1960s, initially had limited applications owing to their low brightness and color rendering. However, with the development and performance improvement of blue and white LEDs, along with reductions in their costs, they are being recognized for their advantages in biotechnology and have been considered next-generation light sources [29, 30]. LED technology offers higher energy efficiency than traditional lighting, enables the precise control of specific wavelengths, and minimizes heat emissions, making it beneficial for plant growth applications [31].

The use of LEDs in plant and callus cultures demonstrates significant potential for controlling growth, development, and metabolism. By adjusting the specific wavelength and light intensity, the content of the desired compounds can be increased, making LED irradiation a valuable method [32-34].

In the present study, AD callus were cultured under blue LED light to investigate their anti-inflammatory activity. Through this study, we aim to highlight the stable production and utilization of natural anti-inflammatory materials.

## Materials and Methods

### Callus Cultivation and Extract Preparation of *Ajuga decumbens* Thunb.

*Ajuga decumbens* Thunb. (AD) callus used in this study were provided by the Biological Resource Center of the Korea Research Institute of Bioscience and Biotechnology. For efficient growth regulation, a medium was prepared containing 0.43% (w/v) Murashige & Skoog medium, 0.01% (w/v) myo-inositol, 3% (w/v) sucrose, 0.4%(w/v) gelrite (all purchased from Duchefa Biochemie, Netherlands), and 0.00001% (w/v) a-naphthaleneacetic acid (purchased from Sigma-Aldrich, USA). The callus was cultured at 25 ± 1°C with a subculture interval of one month.

To evaluate the effect of LED light on callus growth, the samples were cultivated for 10 weeks under blue light (450 ± 50 nm), whereas the control group was maintained under dark conditions. For extraction, 10 volumes of distilled water were added to 1 g of callus, followed by hot water extraction at 70°C. The residual material, excluding the extract, was filtered through paper filters (ADVANTEC, Japan) and subsequently freeze-dried for use in the experiments.

### LC-MS/MS Analysis of *Ajuga decumbens* Callus Extract

The samples were analyzed using liquid chromatography-tandem mass spectrometry (LC-MS/MS) using a Q Exactive UHMR Hybrid Quadrupole-Orbitrap Mass Spectrometer (Thermo Fisher Scientific, USA) coupled to an ultra-high-performance liquid chromatography system (Vanquish Flex UHPLC System, Thermo Fisher Scientific). Solvents A (acetonitrile containing 0.1% (v/v) formic acid) and B (water containing 0.1% (v/v) formic acid) were used as the mobile phases, with a flow rate of 0.3 ml/min. The column used for the analysis was an ACQUITY UPLC BEH C18 column (100 × 2.1 mm, Agilent, USA). The solvent gradient was run over 15 min as follows: 0.0–10.0 min, 10–100% solvent A; 10.0–12.5 min, 100% solvent A; 12.5–12.6 min, 100–10% solvent A; 12.6–15.0 min, 10% solvent A. Each sample was injected at a volume of 5 μl. Full MS spectra in the mass range of 100 to 1,500 m/z were acquired in the positive ionization mode at a resolution of 70,000 FWHM. MS/MS fragmentation spectra were obtained in the data-dependent scan mode using a collision energy of 30 V and a resolution of 17,500 FWHM. The LC-MS data for blue LED-cultured *Ajuga decumbens* callus extract (ADCB) and dark-cultured extracts (ADC) were converted into a global natural product social molecular networking (GNPS)-compatible format (.mzXML) using the GNPS Vendor Conversion Tool and the WinSCP file transfer protocol client. During the filtering process on the GNPS platform, molecular networks were generated by excluding all MS/MS fragment ions within 17 Da of the precursor m/z. MS/MS fragment ion tolerance and precursor ion mass tolerance were set to 2.0 Da and 0.5 Da, respectively.

### Cell Culture

The macrophage cell line, RAW 264.7, used in this study was obtained from the Korean Cell Line Bank (Republic of Korea). The cells were cultured in Dulbeccós modified Eagle’s medium (DMEM, Welgene, Republic of Korea) containing 10% fetal bovine serum (FBS, Welgene), 100 U/ml penicillin, and 100 μg/ml streptomycin (Welgene) at 37°C in a 5% CO_2_ incubator (BB15 CO_2_ incubator, Thermo Fisher Scientific). The cells were subcultured upon reaching 80 – 90% confluence and used for subsequent experiments.

### Cell Viability Assay

The viabilities of cells treated with ADCB and control ADC were evaluated using the thiazolyl blue tetrazolium bromide (MTT) method [35]. RAW 264.7 cells were seeded at a density of 8 × 10^4^ cells/well in 24-well plates and pre-incubated at 37°C under 5% CO_2_ for 24 h. Cells were then treated with ADCB or ADC at concentrations of 25, 50, and 100 μg/ml, along with 1 μg/ml LPS, for 24 h. MTT (1 mg/ml; Sigma-Aldrich) was added to each well and incubated for 2 h. After removing the medium, the formazan crystals that formed were dissolved in DMSO (Sigma-Aldrich, USA). Absorbance was measured at 560 nm using a microplate reader (Thermo Fisher Scientific).

### Determination of NO Production

The NO production induced by LPS in RAW 264.7 macrophages was assessed using the Griess reaction. RAW 264.7 cells were seeded at a density of 8 × 10^4^ cells/well in 24-well plates and pre-incubated at 37°C under 5% CO_2_ for 24 h. The cells were treated with ADCB or ADC at concentrations of 25, 50, and 100 μg/ml, along with 1 μg/ml LPS, and incubated for 24 h. The culture supernatants were mixed with an equal volume of Griess reagent (Sigma-Aldrich), and the inhibitory activity on NO production was measured using an UV/Vis microplate spectrophotometer (Multiskan GO, Thermofisher).

### Determination of PGE_2_, TNF-α, IL-1β, and IL-6 Production

RAW 264.7 cells were seeded at a density of 8 × 10^4^ cells/well in 24-well plates and pre-incubated at 37°C under 5% CO_2_ for 24 h. Cells were then treated with ADCB at concentrations of 25, 50, and 100 μg/ml along with 1 μg/ml LPS and incubated for 24 h under the same conditions. The culture supernatants were collected, and the levels of PGE_2_ and pro-inflammatory cytokines (TNF-α, IL-1β, and IL-6) were measured using enzyme-linked immunosorbent assay (ELISA) kits, including the Mouse PGE_2_ ELISA kit (R&D Systems Inc., USA), Mouse TNF-α ELISA kit (Invitrogen, USA), Mouse IL-6 ELISA kit (BD Biosciences), and Mouse IL-1β ELISA kit (R&D Systems, Inc.).

### Western Blot Analysis

The inhibitory effects of ADCB and ADC on the protein expression of inflammatory mediators were evaluated using western blotting [36]. Antibodies used in this study included iNOS antibody (AHP2399, Bio-Rad, USA), anti-rabbit IgG (#7074) and anti-mouse IgG (#7076), COX-2 antibody (#4842), p44/42 MAPK (Erk1/2) antibody (#9102), phospho-p44/42 MAPK (Erk1/2) (Thr202/Tyr204) antibody (#9101), phospho-SAPK/JNK (Thr183/Tyr185) antibody (#9251), SAPK/JNK antibody (#9252), p38 MAPK antibody (#9212), and phospho-p38 MAPK (Thr180/Tyr182) antibody (#9211) (all from Cell Signaling Technology Inc., USA). Following the same treatment protocol as described above, the cells were lysed using RIPA buffer (Biosesang, Republic of Korea) containing 1 mM phenylmethylsulfonyl fluoride, 0.5 mM Na_3_VO_4_, and 1% protease inhibitor (Sigma-Aldrich). The protein lysates were quantified using the BCA protein assay kit (Thermo Fisher Scientific) and diluted in a 1:1 ratio with 2× Laemmli sample buffer (Bio-Rad), then heated at 100°C for 5 min to prepare the loading samples. Samples were subjected to electrophoresis on 10% sodium dodecyl sulfate–polyacrylamide gels and transferred onto polyvinylidene difluoride (PVDF) membranes (Bio-Rad). The membranes were blocked at room temperature for 2 h with Tris-buffered saline containing 1% Tween 20 (TBS-T) and 5% skim milk, washed with TBS-T, and incubated overnight at 4°C with primary antibodies diluted at a ratio of 1:1000 in TBS-T. After washing, the membranes were incubated with secondary antibodies at room temperature for 2 h and then washed thoroughly with TBS-T. The protein bands were visualized using an ECL kit (Bio-Rad) and an imaging densitometer (LAS-4000, FUJIFILM, Japan). Band intensities were quantified using ImageJ software (NIH, USA) and expressed as graphs.

### Human Primary Skin Irritation Test

A primary skin irritation test was conducted by Korea Dermatology Research Institute Co., Ltd. (Republic of Korea) on healthy volunteers without acute or chronic systemic diseases, including skin disorders. All the participants voluntarily provided written informed consent before the test, which was performed in compliance with the ethical principles of the Declaration of Helsinki. ADCB solutions at concentrations of 50 and 100 μg/ml were prepared in distilled water, and distilled water was used as the negative control. The evaluation was performed according to the criteria of the International Contact Dermatitis Research Group (ICDRG) and the Safety Assessment Guidelines of the Personal Care Products Council (PCPC). The skin irritation index was calculated, and the degree of irritation was determined by referring to the Draize Dermal Classification System (IRB No: KDRI-IRB-240660).

$$\text{Irritation Index =}\frac{\sum \text{Irritation scor at 24,48 and 72h}}{\text{total number of observations}}$$

### Statistical Analysis

Results are expressed as the mean ± standard deviation (SD). The statistical significance of the differences was evaluated using Student’s *t*-test for the data acquired.

## Results

### Changes in Callus Induced by Monochromatic LED Light

**Fig. 1:**
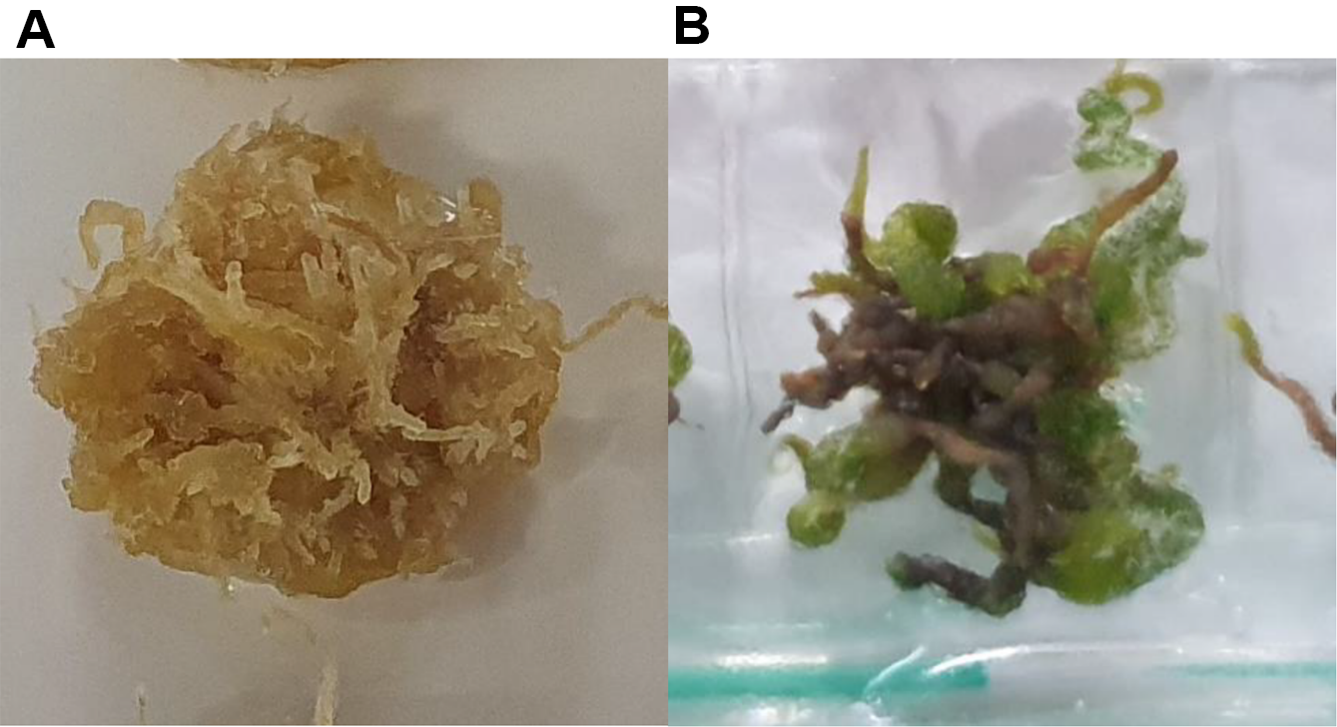
Change of callus colors in light conditions. (**A**) Callus cultured in the absence of light; (**B**) Callus cultured in LED blue light.

**Fig. 2:**
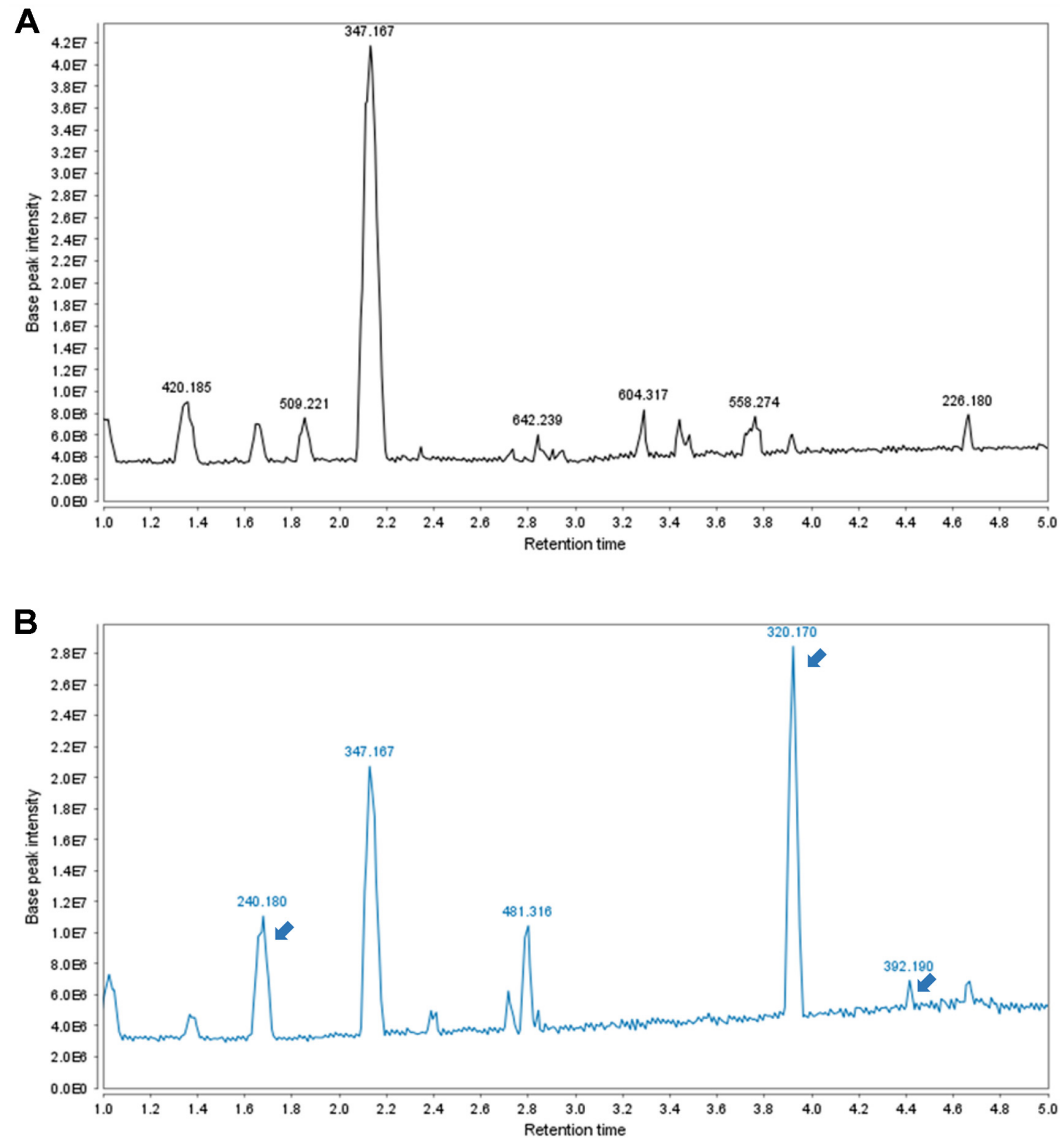
Liquid chromatography-tandem mass spectrometry (LC-MS/MS) analysis of (A) ADC and (B) ADCB. Compounds with various m/z values present in the ADC and ADCB are displayed at the peaks. The three peaks indicated by arrows were specifically observed in ADCB. The m/z value of 392 was detected exclusively in ADCB.

Plants possess photoreceptors such as cryptochrome, phototropin, and zeaxanthin that enable them to sense and respond to various wavelengths of light, which regulate numerous aspects of growth and development [37, 38]. The use of LEDs to manipulate these responses induces growth and diverse reactions in plants, thereby modulating the secondary metabolites [39, 40]. When AD callus was cultured under monochromatic blue LED light and dark conditions, a visual observation revealed that ADCB exhibited a greenish color compared to ADC, and its proliferation was faster (Fig. 1). Additionally, the analysis of secondary metabolite changes under different growth conditions using LC-MS/MS identified four peaks with different profiles in ADCB compared to those in ADC (Fig. 2). Among these, peaks with m/z values of 240 and 420 exhibited increased content, whereas peaks with m/z values of 481 and 392 were observed exclusively in ADCB. These peaks were compared and analyzed with compounds reported to be present in *Ajuga decumbens* through the Reaxys database, and no matching compounds were identified (Supplemental S1). Compounds that increased in ADCB compared to ADC should be isolated and purified, and further studies are required to identify the components whose synthesis is enhanced by blue light.

### Cell Cytotoxicity Measurement

**Fig. 3:**
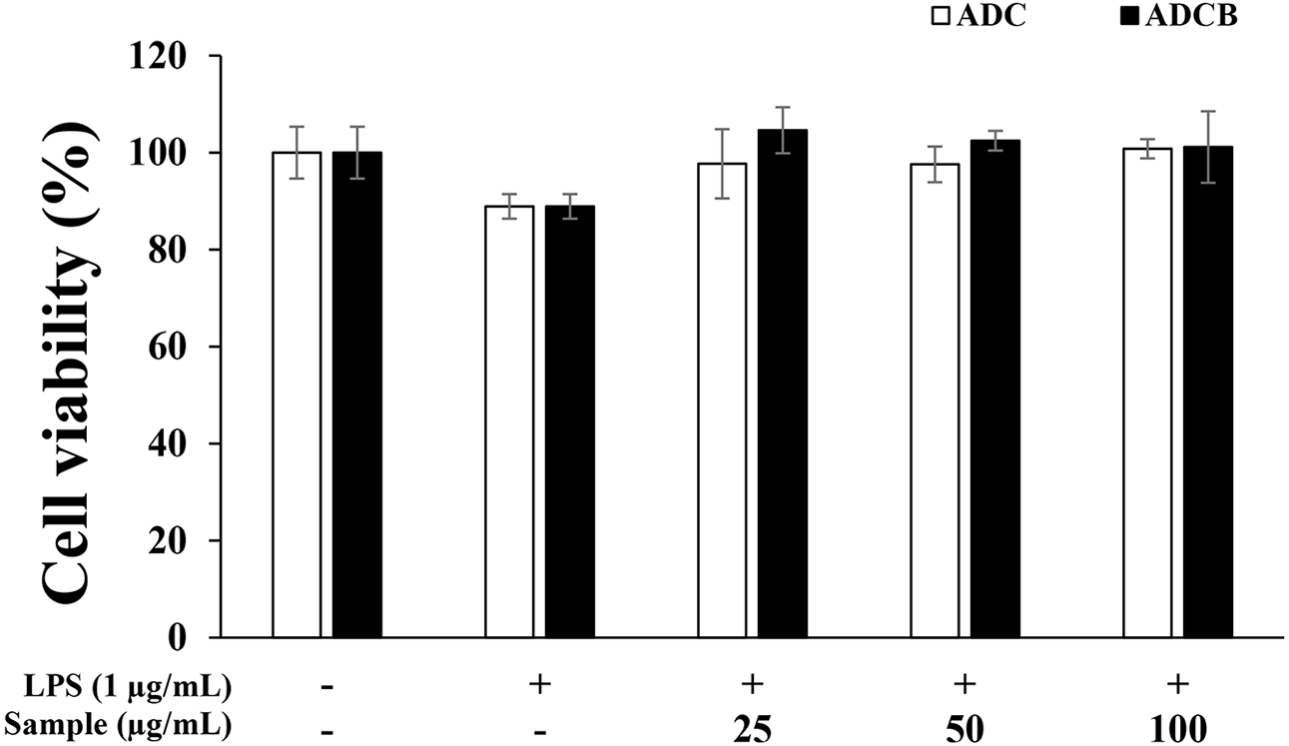
Cell viability of ADC and ADCB in LPS-stimulated RAW 264.7 cell by the MTT assay. The cytotoxicity was determined from the cells induced with LPS (1 μg/ml) in the presence of ADC and ADCB (25, 50, 100 μg/ml) for 24 h.

The viabilities of cells treated with ADCB and its dark-cultured counterpart (ADC) were evaluated using the MTT assay, which measures the reduction of tetrazolium salts to insoluble formazan by mitochondrial reductase enzymes [41]. The results indicated that the ADCB treatment groups demonstrated viability levels of 104.6%, 102.5%, and 101.1% compared with the untreated group, whereas the ADC treatment groups exhibited 99.7%, 97.6%, and 100.1% viability (Fig. 3). Both extracts showed cell viability levels exceeding 97% at all tested concentrations, confirming the absence of cytotoxicity to macrophages. Based on these findings, subsequent experiments were conducted at concentrations that did not exhibit cytotoxicity.

### NO Inhibitory Activity

**Fig. 4:**
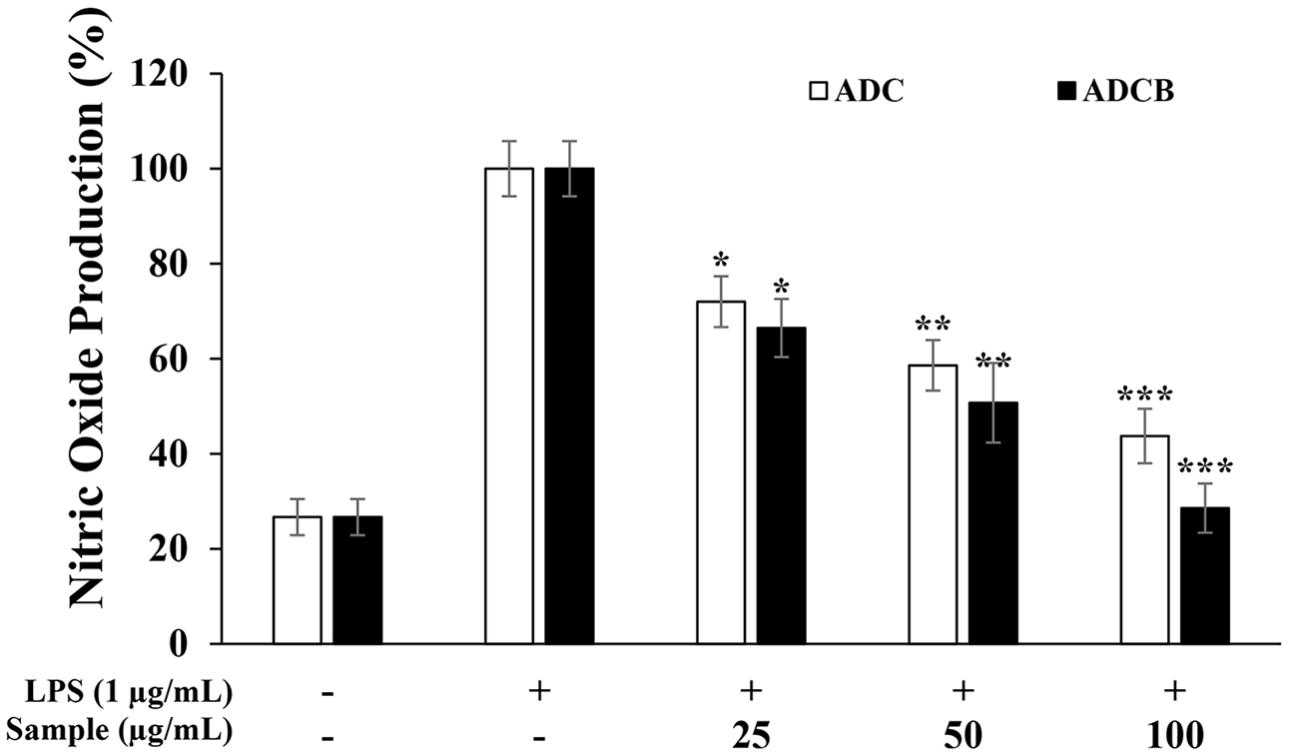
Inhibitory effects of ADC and ADCB on nitric oxide production in RAW 264.7 cell. The production of nitric oxide was determined from the cells stimulated with 1 μg/mL of LPS in the presence of ADC and ADCB (25, 50 and 100 μg/ml) for 24 h. Values represent the mean ± SD with three independent experiments. *p < 0.05; **p < 0.01; ***p < 0.005 versus LPS-only group.

Because NO is a marker of inflammatory responses, its inhibition was measured using an NO assay. This assay quantifies the degradation product of NO, nitrite (NO_2_^-^), which reacts with sulfanilamide to form diazonium salts through the Griess reaction, subsequently forming azo dyes upon coupling with N-(1-naphthyl) ethylenediamine, with absorbance used to quantify NO production [42]. ADCB exhibited NO inhibition rates of 66.5%, 50.7%, and 28.6% across the tested concentrations, compared to the LPS-treated control. ADC exhibited inhibition rates of 72.0%, 58.6%, and 43.7% (Fig. 4). These results suggest that ADC exhibits concentration-dependent anti-inflammatory effects, whereas ADCB, enriched with secondary metabolites from LED cultures, demonstrated enhanced NO inhibitory activity.

### PGE_2_ Inhibitory Activity

**Fig. 5:**
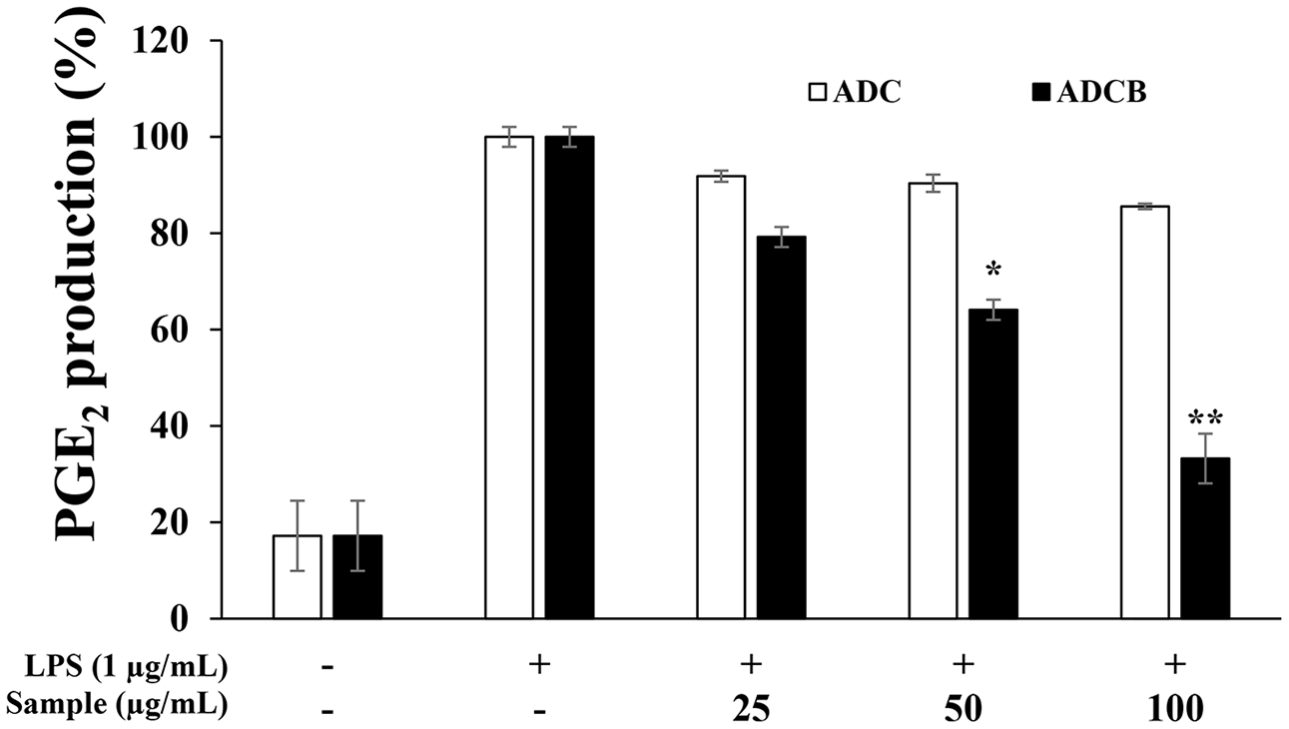
Inhibitory effect of ADC and ADCB on prostaglandin E_2_ production in RAW 264.7 cells. The cells were treated with 1 μg/ml LPS in presence or absence of ADC and ADCB at the indicated concentrations for 24 h. PGE_2_ production was measured by ELISA method from the culture medium of cells. Results are expressed as a percentage of the LPS-only group. Data represent the means ± SD with three separate experiments. *p < 0.05; **p < 0.01 versus LPS-only group.

PGE_2_ is synthesized when COX-2 converts arachidonic acid into prostaglandin H_2_ (PGH_2_), which is then converted to PGE_2_ by PGE synthase. Overproduction of PGE_2_ in inflammatory diseases such as rheumatoid arthritis contributes to cartilage and tissue damage, as well as tumorigenesis [43]. ADCB showed concentration-dependent inhibition of LPS-induced PGE_2_ production, with a maximum inhibition rate of 70.3% at 100 μg/ml compared to the LPS-only group (Fig. 5). In contrast, ADC exhibited a less pronounced inhibitory effect. Thus, ADCB effectively inhibits both NO and PGE_2_ production, indicating its potential as an anti-inflammatory agent capable of restoring inflammatory responses to normal levels.

### Inhibitory Effects on iNOS and COX-2 Protein Expression

**Fig. 6:**
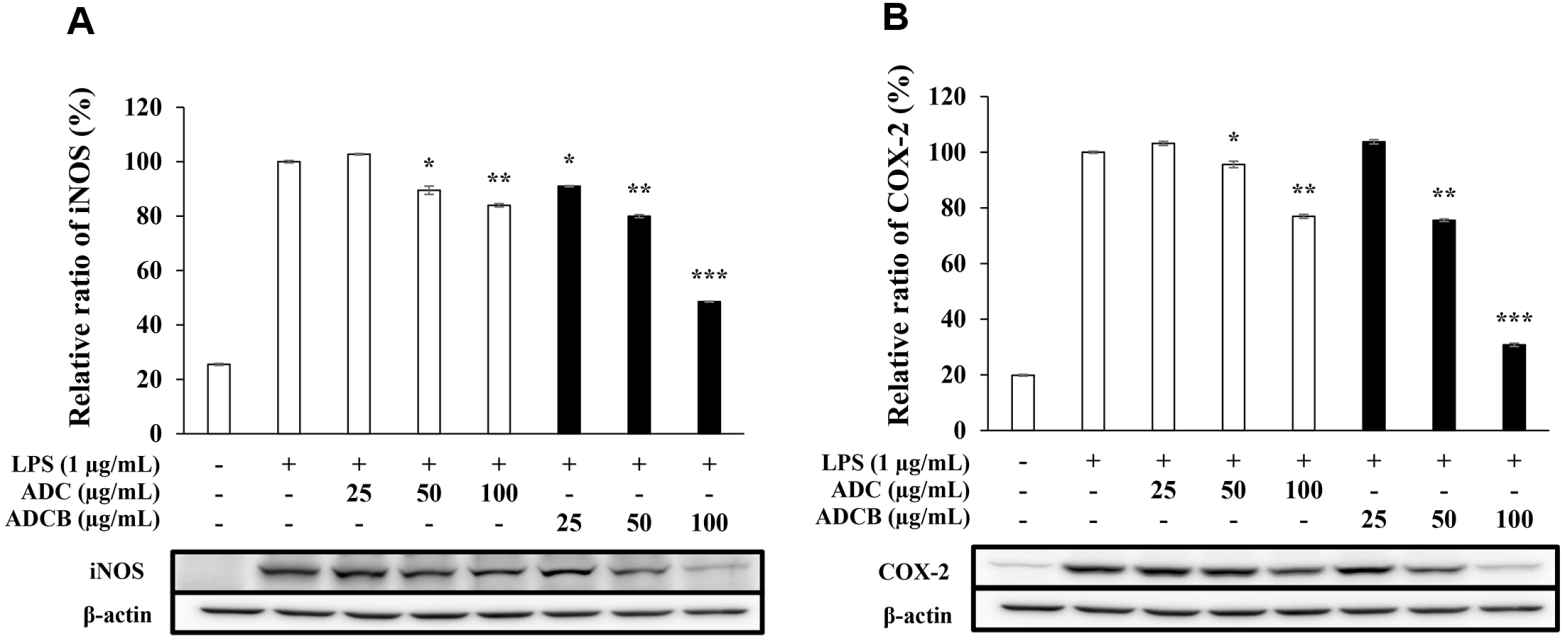
Inhibitory effects of ADC and ADCB on the (A) iNOS protein and (B) COX-2 protein expression in RAW 264.7 cells. The cells were pre-incubated for 24 h, then treated with 1 μg/ml of LPS and indicated concentrations of ADC and ADCB for 24 h. The protein level was analyzed by western blotting. Level of β-actin expression was used as the control. Values represent the means ± SD with independent triplicate experiments. *p < 0.05; **p < 0.01; ***p < 0.005 versus LPS-only group.

Inflammatory stimuli such as interferons and LPS activate iNOS and COX-2, which mediate the production of inflammatory mediators. iNOS catalyzes the prolonged production of NO from L-arginine, whereas COX-2 converts arachidonic acid into PGE_2_. Overexpression of these enzymes exacerbates inflammation [44, 45]. Western blot analysis revealed a concentration-dependent inhibition of iNOS and COX-2 protein expression by ADCB and ADC (Fig. 6). At the highest concentration (100 μg/ml), ADCB inhibited iNOS expression by approximately 51.4% (Fig. 6A) and COX-2 by 69.2% (Fig. 6B), whereas ADC inhibited iNOS and COX-2 expression by 16% and 23%, respectively. These findings confirmed that ADCB was more effective than ADC at suppressing iNOS and COX-2, thereby mitigating excessive inflammation through NO and PGE_2_ regulation.

### Inhibitory Effects on Pro-Inflammatory Cytokine Production (TNF-α, IL-6, and IL-1β)

**Fig. 7:**
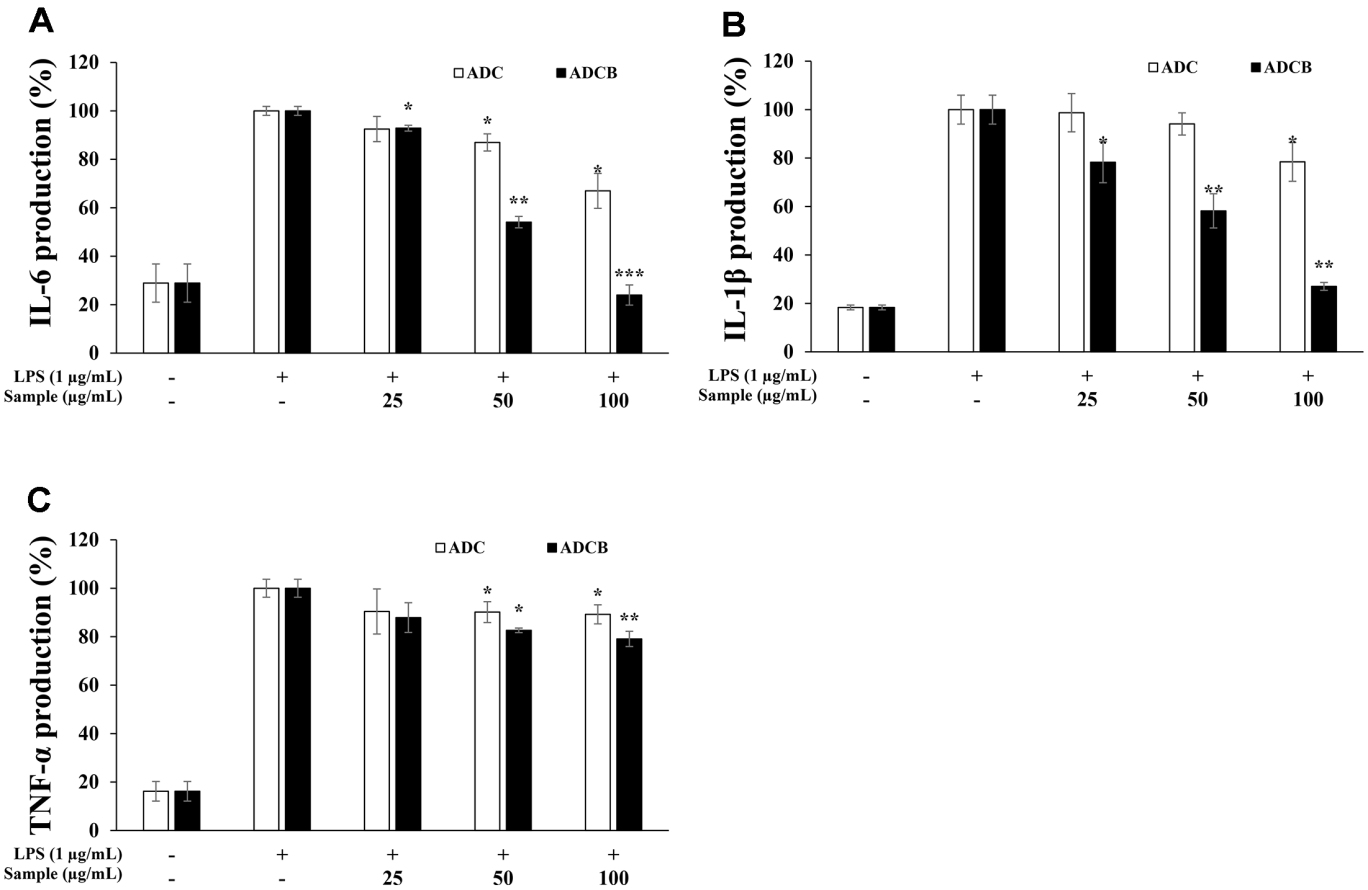
Inhibitory effect of ADC and ADCB on (A) IL-6, (B) IL-1β and (C) TNF-α production in RAW cells. The cells were treated with LPS (1 μg/ml) or with LPS and samples. The production was assayed by ELISA method in the culture medium of cells. The result represent the means ± SD with three separate experiments. *p < 0.05; **p < 0.01; ***p < 0.005 versus only-LPS-treated group.

Among the pro-inflammatory cytokines produced during inflammatory responses, IL-6 induces fever associated with tissue damage, IL-1β is involved in the COX-2 induction process and lymphokine secretion, and TNF-α not only promotes IL-6 expression but is also known to be elevated in diseases accompanied by inflammation and tumor development [46-48]. The inhibitory activities of these cytokines were assessed using an ELISA kit. The results showed that ADCB inhibited their production in a concentration-dependent manner (25, 50, and 100 μg/ml) (Fig. 7). Specifically, TNF-α was inhibited by approximately 12.1%, 17.4%, and 20.9% at each concentration (Fig. 7A), whereas IL-1β was inhibited by 21.7%, 41.8%, and 73.0%, respectively (Fig. 7B). Notably, the IL-6 inhibition rates were 7.1%, 45.9%, and 76.0%, respectively, with the highest concentration demonstrating suppression comparable to that in the untreated group (Fig. 7C). In contrast, ADC inhibited pro-inflammatory cytokines in a concentration-dependent manner; however, its effects were significantly weaker than those of ADCB. This suggests that ADCB can effectively suppress the excessive expression of pro-inflammatory cytokines, potentially regulating inflammatory responses at an appropriate level.

### Expression of Proteins Related to the MAPK Pathway

**Fig. 8:**
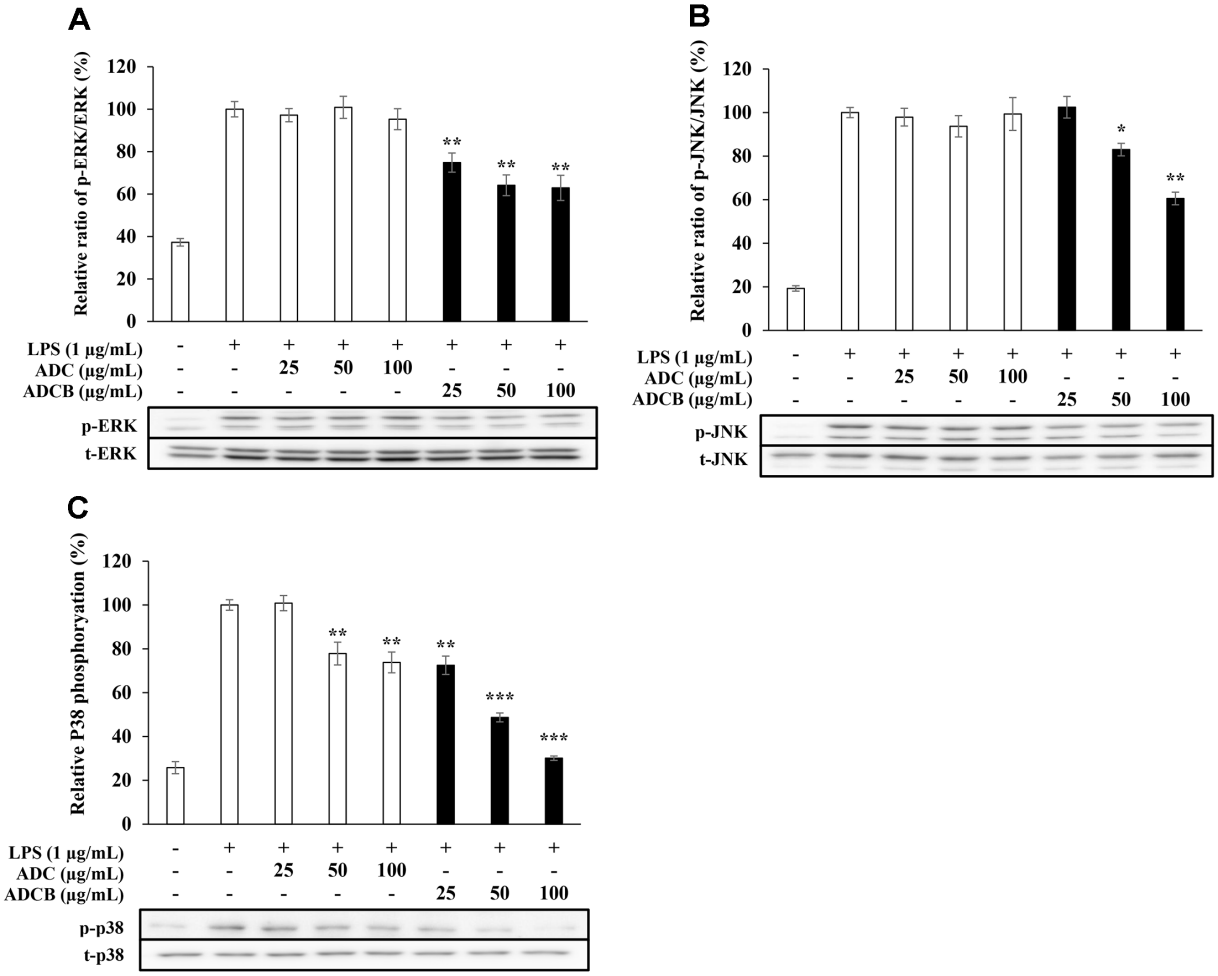
Expression and phosphorylation levels of (A) ERK, (B) JNK and (C) p38 in RAW 264.7 cells. The cells were treated with LPS (1 μg/ml) or with LPS and samples (25, 50 and 100 μg/ml) for 40 min were detected by Western blot analysis. The result represent the means ± SD with three separate experiments. *p < 0.05; **p < 0.01; ***p < 0.005 versus only- LPS-treated group.

To evaluate the inhibitory activity on MAPK phosphorylation, protein expression levels were measured using western blotting. ADCB inhibited ERK phosphorylation by approximately 33%, 42%, and 47% at the tested concentrations (25, 50, and 100 μg/ml), whereas ADC exhibited inhibitory effects of 18%, 13%, and 22% at the same concentrations (Fig. 8A) Furthermore, the phosphorylation of JNK was inhibited by ADCB at levels of -2%, 17%, and 39%, whereas ADC increased phosphorylation in a concentration-dependent manner, with levels of 7%, 3%, and -3%, respectively (Fig. 8B). In the case of P38, ADCB exhibited concentration-dependent inhibition of phosphorylation at 34%, 57%, and 74%, respectively, demonstrating exceptional activity at the highest concentration compared to that of the control group. In contrast, the ADC showed lower inhibitory activity, with inhibition levels of 13%, 31%, and 32%, respectively (Fig. 8C).

These results suggest that the inhibitory effect of ADCB on the expression of various inflammatory mediators is mediated via the MAPK signaling pathway.

### Safety of ADCB Demonstrated through Human Skin Primary Irritation Tests

The safety of ADBC was assessed using a human skin primary irritation test. ADCB at concentrations of 50 and 100 μg/ml was applied to the skin of 30 volunteers using Finn chambers for 24 h. Observations at 30 min, 24 h, and 48 h after removal revealed no skin irritation at either concentration (Table 1, IRB Approval Number: KDRI-IRB-240660).

## Discussion

This study investigated the anti-inflammatory activity of *Ajuga decumbens* cultured under tissue culture and blue LED monochromatic light to overcome the limitations of plant-derived functional materials and to enhance their practical value. LED light sources play a critical role in the activation of photoreceptors that regulate plant growth and metabolism. Blue light activates cryptochromes and phototropins that are pivotal for plant growth, development, and metabolic pathways [49-51]. In this study, the activation of photoreceptors by blue light and the subsequent regulation of metabolic processes induced changes in the synthesis of three secondary metabolites that were undetectable in ADC cultured under standard conditions (Fig. 2). These findings suggest that activation of photoreceptors by blue light promotes the synthesis of secondary metabolites in ADCB.

In experiments evaluating the inhibition of NO and PGE_2_ production to assess the anti-inflammatory activity, ADCB demonstrated significantly greater inhibitory effects than ADC. These results suggest that the compounds present in ADCB effectively regulate the production of inflammatory mediators. Notably, ADCB inhibited NO production in a concentration-dependent manner compared to the LPS-treated group (Fig. 4) and showed excellent inhibitory effects on PGE_2_ production at higher concentrations (Fig. 5). This indicates that ADCB may alleviate inflammatory responses by modulating the excessive production of NO and PGE_2_.

In addition, ADCB inhibited iNOS and COX-2 protein expression to a greater extent than ADC, demonstrating that ADCB fundamentally suppressed the production of inflammatory mediators, such as NO and PGE_2_ (Fig. 6). ADCB also showed superior inhibitory activity at higher concentrations compared to ADC in suppressing the expression of pro-inflammatory cytokines (TNF-α, IL-6, and IL-1β). In particular, the expression levels of IL-6 and IL-1β were suppressed to levels comparable to those of the untreated group (Fig. 7). These results suggested that ADCB effectively regulated the overexpression of pro-inflammatory cytokines, contributing to the potential improvement of inflammatory diseases.

The MAPK signaling pathway plays a critical role in inflammatory responses through the activation of ERK, JNK, and p38, which induce the expression of inflammatory cytokines [52]. In this study, ADCB inhibited the phosphorylation of ERK, JNK, and p38 in a concentration-dependent manner, demonstrating that the anti-inflammatory effects of ADCB are mediated by modulation of the MAPK signaling pathway (Fig. 8). Notably, ADCB exhibited outstanding activity in inhibiting p38 phosphorylation, reducing it to levels comparable to those in the control group at the highest concentration tested. These findings strongly suggest that ADCB is an effective agent for modulating inflammatory responses.

Lastly, a primary skin irritation assessment demonstrated that ADCB at concentrations exhibiting anti-inflammatory efficacy did not induce irritation in human skin. This indicates that ADCB is a safe material with high potential for use in anti-inflammatory cosmetics or pharmaceutical applications.

In conclusion, this study suggests that culturing ADC under blue LED monochromatic light likely activates photoreceptors and enhances the synthesis of specific secondary metabolites, thereby influencing their anti-inflammatory activity. These findings underscore the potential of tissue culture and LED-based cultivation technologies to enable a stable supply of plant resources and increase the content of bioactive compounds, thereby enhancing the utility of natural products. The ability of ADCB to exhibit anti-inflammatory activity without causing skin irritation suggests its significant potential for application in cosmetics and skin disease therapeutics.

This study evaluates the potential of ADCB as a natural anti-inflammatory agent and highlights its possibilities for related research and industrial applications. However, to overcome the limitations of this study, future research should focus on isolating and identifying the specific compounds responsible for the anti-inflammatory activity of ADCB. Additionally, a more in-depth investigation is required to understand the effects of photoreceptor activation on the biosynthesis and metabolic pathways of secondary metabolites in plants. Furthermore, for ADCB to be utilized as a functional anti-inflammatory substance, efficacy assessments using *in vivo* models and long-term safety evaluations are necessary.

## Figures and Tables

**Table 1:** The results of the primary skin irritation evaluation of ADBC.

No.	Test sample	Freauency of skin reaction						Reaction grade (R)*	
		30 min after removal		24 h after removal		48 h after removal		Irritation index	Judgment
		0.5	0	0.5	1	0.5	1		
1	ADCB 50 ppm	1	-	-	-	-	-	0.006	Non
2	ADCB 100 ppn	2	-	1	-	-	-	0.017	Non
3	Purified water	-	-	-	-	-	-	0.00	Non

The reactions were evaluated at 30 min, 24, and 48 h after the samples were removed in accordance with the ICDRG criteria and PCPC guidelines.

*The range of non-irritation: 0 ≤ R < 0.02
